# Intellectual and school performance evaluation of children submitted to tonsillectomy and adenotonsillectomy before and after surgery

**DOI:** 10.1590/S1808-86942012000400005

**Published:** 2015-10-20

**Authors:** Felipe Hideo Ikeda, Patricia A. de Campos Horta, Wilze Laura Bruscato, José Eduardo Luftaif Dolci

**Affiliations:** Specialized in Larynx; ENT Physician; Psychoanalyst (Hospital Psychologist - Psychology and Pediatrics Department of the Irmandade da Santa Casa de Misericórdia de São Paulo Hospital); PhD (Manager of the Psychology Department - Irmandade da Santa Casa de Misericórdia de São Paulo Hospital; Adjunct Professor - Medical Sciences School - Santa Casa de São Paulo Hospital); PhD (Director of the Medicine Program and Full Professor at the Medical Sciences School - São Paulo

**Keywords:** performance tests, child, palatine tonsil, adenoids

## Abstract

Several studies have demonstrated the impact of obstructive sleep disorders on the quality of life of children. However, few studies address school and intellectual performances of children who have undergone tonsillectomy or adenotonsillectomy, indicating the benefits of these surgeries.

**Objective**: To evaluate and compare the learning and intellectual performances of children submitted to tonsillectomy or adenotonsillectomy, before and after surgery.

**Materials and Methods**: 83 children between the ages of 7 and 11 were evaluated by a psychologist employing a longitudinal and descriptive study in the pre and post-surgery groups. The first evaluation was performed just before surgery, and the second and third evaluations one and six months after the surgical procedure. The social-demographic form, Raven's Colored Progressive Matrices Test and the School Performance Test were used.

**Results**: The group of children in this study presented a statistically significant evolution in their intellectual performance evaluations (*p* < 0.05) and also school performance evaluations in writing, mathematics and reading sub-tests (*p* < 0.001).

**Conclusion**: Based on our findings we concluded that tonsillectomy or adenotonsillectomy performed in children with obstructive respiratory disorders produce a positive impact on intellectual and school learning development.

## INTRODUCTION

Difficulties in school performance are almost always associated with different problems, emotional or family issues, or even to diseases. What frequently happens is that children with such difficulties are described as having little dedication to school activities^1^. Nonetheless, it is not uncommon to find intelligence levels matching those, or even higher, of other children in the same age range^2^.

The low intellectual and school performance may bring suffering to the child, low confidence in his potentials and feelings of failure which may affect his self-esteem, thus bringing about repercussions in his entire development, and also to the lives of family members^3^.

Pharyngeal and palatine tonsil hyperplasias are frequent causes of nasal obstruction and chronic oral breathing during childhood - becoming the main breathing obstruction disorder, which may cause numerous clinical changes, from apnea - with and without cardiopulmonary repercussions, all the way to changes in craniofacial development, postural changes, atypical deglutition, poor feeding, and others. Because of these symptoms the child may also have school learning disorders - which is not well clarified in scientific research, but rather by deduction based on the physician's experience and reports from patients and parents.

Tonsillectomy and adenotonsillectomy remain the most frequently performed surgical procedures in otorhinolaryngology. In properly selected children, they may change the quality of life perception and solve the obstructive symptoms^4^.

Uema et al.^5^ carried out learning tests in children with sleep obstructive apnea syndrome (SOAS), previously tested with polysomnography and allocated them in one of three groups: SOAS, primary snoring and children without sleep obstructive disorders. The results showed statistically significant differences in the SOAS group and primary snoring when compared to the control group. In this study, we tested the immediate memory and attention level (which impacts memory processing), which proved to be impaired in the group of patients with obstructive sleep disorders, since these evocations are directly associated to the level of attention and remembering the information presented once only - immediate memory.

Silva & Leite^6^ carried out an interventional study, before and after surgery, in which they assessed 48 children, with mean age of 5.93 years, with indication of tonsillectomy or adenotonsillectomy at an ENT outpatient ward, and guardians received the Obstructive Sleep Apnea (OSA - 18) questionnaire, specific to assess quality of life before surgery and with a minimum of 30 days afterwards. They carried out a nasal fibroscopic exam, complete ENT physical exam and a semi-structured questionnaire on the clinical and social profiles of the child, in both consultations. The most frequent symptoms were: restless sleep, apnea and snoring. Sleep obstructive disorders had a relevant impact on quality of life and improved considerably after surgery.

Goodwin et al.^7^ performed 480 polysomnography in children of 6 to 12 years of age, of both genders, with SOAS and found significant associations between the symptoms and polysomnographic findings, including their rate of attention and learning.

In another study, Gozal & Pope^8^ investigated 300 children with the worst grades in a school, 18.1% of them had gas-exchange (O_2_ and CO_2_) disorders during sleep. Of those with sleep obstructive disorders, half were submitted to adenoidectomy. They noticed that the group submitted to adenoidectomy had a significant increase in the aforementioned mean value. The other half, without disorders, kept their mean scores.

Ezzat et al.^9^ analyzed the intelligence coefficient of 84 children with sleep apnea syndrome before and after they were submitted to adenotonsillectomy and noticed an improvement in the postoperative performance in most of them.

Kim et al.^10^ utilized the information from the parents of 302 children for the diagnosis of sleep apnea syndrome and found clear association with bad school performance in them.

Numerous studies in the international literature^7^, ^11^, ^12^, ^13^ have been showing the impact of sleep obstructive disorders in learning, memory, attention and the very quality of life of these children. Notwithstanding, so far, there are but a few papers published^6^ on the subject. And, in even fewer cases, in national and international studies, the school performance of patients submitted to tonsillectomy or adenotonsillectomy, is assessed; thus causing certain questions about the benefit os such procedure in the education of a population.

The goal of the present paper is to assess and compare the intellectual and school performances of children submitted to tonsillectomy and adenotonsillectomy before and after the procedure.

## MATERIALS AND METHODS

### Study Design

Longitudinal and cross-sectional.

### Sample

In this study, we had 83 patients between 7 and 11 years of age, of both genders, who were submitted to tonsillectomy and adenotonsillectomy surgeries between June of 2009 and April of 2011.

### Inclusion criteria

Patients with surgical indication associated with upper airway obstruction. The decision for surgery was based on the patient's history and physical exam:

• Symptoms during sleep: snoring; excessive movements during sleep; excessive sweating; “suffocation” fits reported by the parents or informants.
• Behavioral disorders: psychomotor restlessness; excessive irritability; tiredness during the day or upon waking up; bad school performance.
• Mouth breathing, nasal voice.
• Physical exam: tonsils grades III and IV, which are considered obstructive according to Brodsky's classification.
• Complementary tests: Cavum x-ray and/or nasal endoscopy, showing an important degree of nasopharyngeal obstruction.

The patients had their surgical indication based on some of the signs and symptoms of the groups aforementioned, and most of them were: snoring, mouth breathing and tonsil hyperplasia.

### Exclusion criteria

• Patients with changes in neurological and psychomotor development;
• Patients with psychiatric and behavioral disorders;
• Patients with syndromes;
• Patients with visual disorders;
• Patients who use medication acting on the central nervous system (CNS);
• Patients with hearing disorders (interview, otoscopy and audiometry);
• Patients with chronic or recurrent pharyngo- tonsillitis;
• Patients with a history of peritonsillar abscess;
• Patients with halitosis or caseous tonsillitis;
• Patients with tonsil asymmetry or possible malignancy;
• Patients with clinical history of upper airway obstruction not matching physical exam findings.

### Instruments

We used the following study instruments:

#### Sociodemographic Form

Created by the researchers with the aim of collecting information about the sociodemographic profile of the patients.

#### *Raven's Test of Progressive Colored Matrices - Special Scale*^*14*^

This is a non-verbal instrument to assess intelligence, validated and adapted to be used in Brazil^15^, which enables the assessment of the general intelligence factor (ability to understand new situations, ability to remember relevant information, judgement and build up of specialized information). This test can be utilized in children between 5 and 11.5 years of age. It is made up of three series (A, Ab and B), with 12 problems in each one, ordered in ascending order of difficulty and which, added together, provide the general score. The solution of their problems requires a spacial construction scheme.

#### *SPT - School Performance Test*^*16*^

This is a standardized psychometric instrument, which aims at assessing school performance. This test can be utilized in children between 7 and 12 years. The SPT is made up of three subtests: written (first name and words alone presented in a dictation), arithmetics (oral solution of problems and calculations of arithmetical operations in written) and reading (recognition of words apart from the context).

### Procedures

Data collection started after the Project was approved by the Scientific Committee, as well as by the Ethics in Research with Human Beings Committee of the Institution (Project $n^{\underline{o}}$ 152/09 CEP).

The patients were assessed by an otorhinolaryngologist and those with surgical indication were referred to the Psychology ward.

The researchers made a brief explanation about the study, as well as the goals and the guardian signed the Informed Consent Form, pertaining to Resolution $n^{\underline{o}}$ 196 of October 10, 1996, from the National Council of Health, after fully understanding and agreeing to participate.

The researchers collected the data in a private psychologist room, which guaranteed secrecy and privacy.

The patients were assessed by the psychology professionals at three moments. In the first assessment (before surgery), the parents filled out the Sociodemographic Form and the children were submitted to the Raven's Progressive Colored Matrices Test and the School Performance Test (SPT) - which aimed at assessing intellectual and school performances, respectively. The patients were then submitted to tonsillectomy and adenotonsillectomy in the Operating Theater under general anesthesia and orotracheal intubation. Afterwards, they were discharged and received post-operative care. Following that, they were referred to ENT outpatient followup and to psychological assessment - six months after the procedure. We decided to do the first postoperative assessment with one month, because that would be when the child had no more pain, swallowing difficulties, fever, headache and others, which could hamper their assessment. In these postoperative evaluations, only the children were submitted to the tests and it took between 60 and 90 minutes to perform.

### Data Analysis

The data obtained was collected and inserted in the database created in the SPSS for Windows version 13.0^17^ to obtain scores and for the descriptive and inferential statistical assessment of the sample.

We used the Anova variance analysis model to compare the scores along time, with repetitive measures. The analyses of the scores between the assessments was carried out by means of multiple comparisons.

The level of significance adopted was 5% (0.05%).

It is worth stressing that for the descriptive analysis of the data we considered 83 patients in the first and 75 in the third assessment. Nonetheless, in order to have statistical significance, the sample had 75 patients in each.

## RESULTS

### Sociodemographic Data

The sample was made up of 83 patients submitted to tonsillectomy and adenotonsillectomy. One hundred and two patients participated in the pre-operative assessment. Of these, eight were not operated (one patient was disconnected from the hospital, two patients gave up on the surgery, two patients had contraindications to the procedure and three patients awaited tests in order to undergo surgery), one patient was taken off the study for having a diagnosis of epilepsy and then patients went through only the first assessment and surgical procedure. Eighty-three patients underwent the first and second assessments and 75 patients went through the first, second and third assessments until the conclusion of the study.

The 83 patients in the sample had a mean age of 8.6, standard deviation (SD = 1.3). Forty-seven patients (56.6%) were male and 36 (43.4%) were female.

Concerning the other sociodemographic data collected, the results are depicted on Table 1.

**Table 1:** Socio-demograpbhic variables of our sample (n=83).

Variables	n (%)
1. Education (1^st^ assessment)	1^st^ grade = 18 (22%) 2^nd^ grade = 21 (25.6%) 3^rd^ grade = 27 (32.9%) 4^th^ grade = 8 (9.8%)5^th^ grade = 7 (8.5%)6^th^ grade = 1 (1.2%)
2) School	Public School = 81 (97.6%) Private School = 2 (2.4%)
3) Skin color	White = 51 (61.4%) Brown = 23 (27.7%) Black = 8 (9.6%) Yellow = 1 (1.2%)
4) Born in5) Education of the mother/guardian	City of São Paulo = 76 (91.6%) Country side of São Paulo = 1 (1.2%) Northeast of the country = 5 (6.0%) Midwest of Brazil = 1 (1.2%)
5) Education of the mother/guardian	Illiterate = 2 (2.4%)Incomplete elementary I = 8 (9.6%) Complete elementary I = 7 (8.4%) Incomplete elementary II = 13 (15.7%) Complete elementary II = 13 (15.7%) Incomplete high school = 10 (12%) Complete high school = 24 (28.9%) Incomplete higher education = 3 (3.6%) Did not know how to answer = 3 (3.6%)
6) Education of the father/guardian	Illiterate = 1 (1.2%)Incomplete elementary I = 10 (12%) Complete elementary I = 6 (7.2%) Incomplete elementary II = 27 (32.5%) Complete elementary II = 5 (6%) Incomplete high school = 7 (8.4%) Complete high school = 20 (24.1%) Incomplete higher education = 1 (1.2%) Complete higher education = 1 (1.2%) Did not know how to answer = 5 (6%)

As far as schooling is concerned, in the first assessment, 18 patients (22%) were in the 1^st^ grade, 21 were in the 2^nd^ grade (25.6%); 27 (32.9%) in the 3^rd^ grade; eight (9.8%) in the 4^th^ grade; seven (8.5%) in the 5^th^ grade and one (1.2%) in the 6^th^ grade. Eighty-one children (97.6%) went to public school and two (2.4%) to private school.

As to skin color, 51 patients (61.4%) were white; 23 (27.7%) were brown; eight (9.6%) were black and one (1.2%) was yellow.

In the first assessment, 67 patients (80.7%) did not have urinary and/or fecal incontinence, but 16 (19.3%) had some type of incontinence. In the second assessment, 75 patients (90.4%) did not have urinary and/or fecal incontinence and eight (9.6%) had some type of incontinence. In the third assessment, of the 75 patients assessed, 69 (92%) did not have urinary and/or fecal incontinence and it was only six (8%) who still had some type of incontinence.

All the patients were Brazilians, 76 (91.6%) were from the city of São Paulo, one (1.2%) from the countryside of the State of São Paulo; five (6.0%) were from the Northeast and one (1.2%) from the Brazilian Midwest.

As to the education of the mothers/guardians, two (2.4%) were illiterate, 15 (18%) had elementary education I - eight (9.6%) had not completed it and seven (8.4%) did; 26 (30.4%) had elementary education II - 13 (15.7%) had not completed it and 13 (15.7%) did; 34 (40.9%) had high school - 10 (12%) did not complete it and 24 (28.9%) did; three (3.6%) had incomplete higher education and three (3.6%) responders did not know how to answer it.

As to the education of the fathers/guardians, one (1.2%) was illiterate; 16 (19.2%) had elementary education I - 10 (12%) did not complete it and six (7.2%) did; 32 (38.5%) had elementary education II - 27 (32.5%) did not complete it and five (6%) did; 27 (32.5) had high school education - seven (8.4%) did not complete it and 20 (24.1%) did; two (2.4%) had higher education - one (1.2%) did not complete it and one (1.2%) finish it. Five (6%) responders did not know how to answer.

### Performance data

#### Raven's Progressive Colored Matrices - Special Scale

##### Results from the Total Number of Points

In assessing intelligence, the total number of points achieved by the patients in the sample in the first assessment had a mean value of 21.2 (SD = 5.9) and median of 21, in the second assessment, the mean value was 22.8 (SD = 6.3) and a median of 23; and in the third assessment, the mean value was 24.7 (SD = 5.8) and median value of 25.

We noticed an evolution in the performance of these patients in the first to second assessment, in the second to the third and in the first to the third assessment (*p* < 0.001).

##### Percentile Result

As to the percentile, the sample had 56.6 (SD = 26.2) as mean percentile, and median value of 60 in the first assessment, mean of the 61.3 percentile (SD = 27.6) and median 70 in the second assessment and percentile 66.5 mean (SD = 24.6) and median 75 in the third assessment, which can be seen on Table 2.

**Table 2:** Results from the Raven Progressive Colored Matrices Test percentiles - Special Scale in the three assessments.

	Assessment	Mean	SD	Median	*P*
Raven	1	56.6	26.2	60.0	*p* < 0.001*
	2	61.3	27.6	70.0	
	3	66.5	24.6	75.0	*p* < 0.001*

SD: Standard Deviation;

* Comparison between the first and the third assessments.

Upon comparing the mean number of points in this score through variance analysis, we found a statistically significant evolution in the performance of these children (*p* < 0.001). Comparing the results of the three assessments, we found a statistically significant difference from the first to the third assessment (*p* < 0.001).

##### Results from the Level of Intelligence

Analyzing the first and third assessments, we noticed that in the first there were 30 (40%) children with higher or above average level of intelligence, 34 (45.3%) on average and 11 (14.7%) below average or intellectually handicap. On the third assessment, 39 (52%) children had higher or above average intelligence level, 30 (40%) were on average and six (8%) were below average or intellectually handicap, which shows the statistically significant evolution in the intellectual performance of these children from the first to the third assessments (*p* = 0.041) according to the Mc Nemar test. Table 3 depicts the comparison of the intellectual performance results from the first to the third assessments.

**Table 3:** Comparison of the Intellectual Performance in the Raven Progressive Colored Matrices - Special Scale from the 1^st^ to the 3^rd^ Assessment

Raven	1^st^ Assessment	3^rd^ Assessment	*p*
Above average and higher	30 (40%)	39 (52%)	*p* = 0.041*
Mean	34 (45.3%)	30 (40%)	
Below average and handicap	11 (14.7%)	6 (8%)	*p* = 0.041*

* Comparison between the first and the third assessments.

#### School Performance Test

##### Writing Subtest

The first writing subtest assessment had a mean score of 14.8 (SD = 10.0) and median of 14; in the second assessment, the mean was 15.7 (SD = 10.1) and median of 16; and, in the third assessment the values were: mean of 17.6 (SD = 9.9) and median of 19.

Comparing the written subtest scores showed a significant evolution in the children (*p* < 0.001).

Upon analyzing the three assessments, we noticed significant differences between the second and the third assessments (*p* < 0.001) and between the first and the third (*p* < 0.001).

##### Arithmetics Subtest

The first assessment of the arithmetics subtest produced a mean value of 10.47 (SD = 6.62) and median value of 9.0; in the second assessment, the mean was 10.75 (SD = 6.84) and median of 9.0; and, in the third assessment, the mean value was 12.23 (SD = 6.82) and median of 11.0.

There was a statistically significant performance evolution in this subtest after surgery (*p* < 0.001). A more detailed investigation showed an evolution from the second to the third assessment (*p* < 0.001) and from the first to the third assessment (*p* < 0.001).

##### Reading Subtest

The first assessment of the reading subtest produced a mean score of 45.8 (SD = 25.3) and median of 58; in the second assessment, the mean was 60.0 (SD = 24.7) and median of 60.0; and, in the third assessment, the mean was 53.7 (SD = 20.8) and median of 65.0.

We also noticed a significant performance evolution in this subtest after surgery (*p* < 0.001): from the first to the second assessment (*p* = 0.001), from the first to the third assessment (*p* < 0.001) and from the second to the third assessment (*p* < 0.001).

The results from the writing, arithmetics and reading sub-tests are depicted on Table 4.

**Table 4:** Summary measures of the School Performance Test (SPT) between the three assessments.

SPT	Assessment	Mean	SD	Median	*P*
Writing	1	14.8	10.0	14.0	*p* < 0.001^1^
	2	15.7	10.1	16.0	*p* < 0.001^2^
	3	17.6	9.9	19.0	*p* < 0.001^1^, ^2^
Arithmetics	1	10.5	6.6	9.0	*p* < 0.001^3^
	2	10.8	6.8	9.0	*p* < 0.001^4^
	3	12.2	6.8	11.0	*p* < 0.001^3^, ^4^
Reading	1	45.8	25.3	58.0	*p* = 0.001^5^, *p* < 0.001^6^
	2	48.6	24.7	60.0	*p* = 0.001^5^, p < 0.001^7^
	3	53.7	20.8	65.0	*p* < 0.001^6^, ^7^
Total	1	71.0	39.4	79.0	
	2	74.9	38.9	86.0	
	3	83.5	34.7	93.0	

SD: Standard Deviation.

1 Comparison between the first and the third assessments;

2 Comparison between the second and the third assessments;

3 Comparison between the first and the third assessments;

4 Comparison between the second and the third assessments;

5 Comparison between the first and the second assessments;

6 Comparison between the first and the third assessments;

7 Comparison between the second and the third assessments.

## DISCUSSION

Difficulties in school performance are frequently a reason for referral to specialists. These can be associated with emotional aspects, family issues or even diseases. Despite being intelligent, many children have learning difficulties.

Respiratory discomfort and sleep apnea are disorders which may also affect a child's school performance, as well as behavior, development and quality of life.

In order to assess whether tonsillectomy and adenotonsillectomy surgeries would bring benefits as far as school performance is concerned, psychological evaluations were carried out before and after the surgery. Through this process, the children in the study group had significant improvements in the intellectual performance test and in the writing, arithmetics and reading tests, which assess school performance.

Thus, similarly to the studies mentioned in the References below, this study also showed significant evidence that treating disorders associated with sleep apnea syndrome in children brings about benefits vis-à -vis their intellectual and school performance.

Uema et al.^5^ proved that children with OSAS and primary snoring had their immediate memory and attention level impaired; as did Goodwin et al.^7^, who found associations between the polysomnographic findings and the learning and attention indices in children with OSAS. Our study showed that, for these patients, the proper treatment of such conditions may bring about benefits for the child's intellectual and school performances, because it enables these children to better utilize their potential and better develop their skills, and this could be seen by the results of the tests carried out and corroborated by the accounts from parents and guardians, who noticed that after the surgical procedure, the children became more attentive and also slept better, without snoring, which benefitted not only the children, but also the entire family.

Gozal & Pope^8^ investigated children with the worst grades in a school, and 18.1% of them had sleep disorders. Half were submitted to adenotonsillectomy and, by assessing their school report cards, they found an improvement in the grades of this group. Comparatively, our study also confirmed this information by means of the specific and standardized tests to assess intellectual and school performance in children.

A recent paper by Kim et al.^10^ confirmed that children from an elementary school in Seul, with obstructive sleep apnea syndrome had low school performance. Another study, by Ezzat et al.^12^, showed an improvement in the intelligence tests of patients when comparing their results before and after adenotonsillectomy. Both papers corroborate the data analysis produced in our study.

Thus, we may stress an improvement in the school and intellectual performances of these children, properly diagnosed and submitted to tonsillectomy and adenotonsillectomy surgeries - which will probably bring about benefits to the quality of life of these children and their families.

During the psychological assessments, in the first evaluations of some children the parents reported some type of incontinence. Nonetheless, we noticed that during the second and third evaluations that some of these children improved and no longer had incontinence. This data require further studies to be properly investigated.

As a limitation of the present study, we stress the lack of a control group with children of the same age range, but without OSAS signs and symptoms, in order to compare and analyze if the the school and intellectual developments of our sample are equal to or lower than that of the control group before and after surgery, and also to take the child's own growth and development process out of the equation.

This study proved the fundamental importance of properly treating the Obstructive Sleep Apnea Syndrome in children. By means of these specific tests, we could show that surgeries used to treat OSAS, when properly indicated, play a most relevant role in the school and intellectual developments of children with such disorder. Thus, this surgical approach deserves more attention from public health authorities, because it may bring about benefits for the school and intellectual performances of children, and, consequently, to the very education of this population.

## CONCLUSION

Assessing intelligence performance in the post-operative of tonsillectomy and adenotonsillectomy, when compared to pre-operative levels, showed a statistically significant evolution in these children in their total scores, in the percentage score and for their intelligence level performance.

When we assessed school performance before and after tonsillectomy and adenotonsillectomy and compared the mean scores throughout three assessments, we noticed better performance after surgery, for each subtest (writing, reading and arithmetics) and for their total score.

The group of children in the present study had a satisfactory evolution in their intellectual performance. Our findings also show that tonsillectomy and adenotonsillectomy surgeries performed in children with obstructive sleep disorders may have a positive impact in their intellectual development and school performance.
